# Virtual-Wall Model for Molecular Dynamics Simulation

**DOI:** 10.3390/molecules21121678

**Published:** 2016-12-09

**Authors:** Lijuan Qian, Chengxu Tu, Fubing Bao, Yonghao Zhang

**Affiliations:** 1 Institute of Fluid Measurement and Simulation, Department of Mechanics Engineering, China Jiliang University, Hangzhou 310018, China; qianlj@cjlu.edu.cn (L.Q.); dingobao@cjlu.edu.cn (F.B.); 2 James Weir Fluids Laboratory, Department of Mechanical and Aerospace Engineering, University of Strathclyde, Glasgow G1 1XJ, UK; yonghao.zhang@strath.ac.uk

**Keywords:** virtual-wall model, molecular dynamics, fluid-wall interaction, surface roughness, nano-scale gas flow

## Abstract

A large number of molecules are usually required to model atomic walls in molecular dynamics simulations. A virtual-wall model is proposed in this study to describe fluid-wall molecular interactions, for reducing the computational time. The infinite repetition of unit cell structures within the atomic wall causes the periodicity of the force acting on a fluid molecule from the wall molecules. This force is first calculated and then stored in the memory. A fluid molecule appearing in the wall force field is subjected to the force from the wall molecules. The force can then be determined by the position of the molecule relative to the wall. This model avoids excessive calculations of fluid-wall interactions and reduces the computational time drastically. The time reduction is significant for small fluid density and channel height. The virtual-wall model is applied to Poiseuille and Couette flows, and to a flow in a channel with a rough surface. Results of the virtual and atomic wall simulations agree well with each other, thereby indicating the usefulness of the virtual-wall model. The appropriate bin size and cut-off radius in the virtual-wall model are also discussed.

## 1. Introduction

Micro/nano-electromechanical systems have received considerable attention over the past several decades. Many of these systems involve components that deal with fluid flows. These components include motors, actuators, sensors, and valves [1,2,3,4]. Understanding the fluid behavior in these systems requires simulation capability at micro/nano length scales, especially when performing experimental measurements is difficult. Therefore, various simulation methods have been developed to capture flow physics at these scales [5,6,7,8,9,10,11].

A distinct feature of fluid flow in micro/nano-scale confined devices is the effect of the wall surface force [12,13,14]. The wall force penetration depth is approximately 1 nm for an atomically smooth wall [15]. With the decrease in the flow characteristic length scale, the surface force serves an increasing important function in fluid flow, e.g., 40% of a 5 nm height channel will be subjected to wall force field effects; under these effects, the momentum and energy transport characteristics of a fluid can significantly deviate from kinetic theory predictions [15]. Therefore, the influence of this near-wall force field on the nano-scale confined flow must be understood and evaluated, and deviations from kinetic theory predictions must be characterized.

Molecular dynamics (MD) simulation studies the physical movements of atoms and molecules through N-body simulation; such simulations can accurately describe the fluid-wall interactions when the potentials between molecules are well defined [16]. This method has been employed by many researchers to investigate the liquid flow in nano-scale-confined channels [17,18,19,20]. Moreover, this method was recently used to study the gas flow in nano-scale channels [15,21,22,23,24].

The main drawback of the MD simulation lies in its computational cost. In confined fluid flows, a large number of molecules are required to model the atomic wall. Such a large number may be significantly larger than that of the fluid molecules. This drawback is particularly fatal for gas flow in confined channels. For example, Barisik et al. [25] reported a nano-scale Couette flow at *Kn* = 10, with a simulation box of 162 nm × 3.24 nm × 162 nm; in the simulation, the number of gas molecules was 4900, and the number of wall molecules was 903,003. Therefore, most of the computational resources were consumed on the computation of wall molecules.

Walls in the MD simulation can be an atomic or idealized model. The atomic nature of a wall is neglected in the ideal wall model. The fluid-wall interactions are often described using rules, such as the diffuse and specular reflections, Maxwell’s scattering kernel [26], or the Cercignani-Lampis model [27]. These ideal wall models are acceptable in specific situations. However, when focusing on the fluid flow characteristics near the wall, the atomic wall model must be employed.

Barisik et al. [25] proposed the smart wall model for handling the large number of wall molecules in nano-scale gas flows. A total of 74 wall molecules were used to construct a 3D face-centered cubic (FCC) wall unit, which was stored and utilized to calculate molecule-surface collisions at each step. Hence, the total computational time could be significantly reduced. However, 74 gas-wall intermolecular forces still needed to be calculated for each gas molecule in the wall force field. When the number of molecules in the wall force field is large (e.g., in high-density gas or liquid flow), the computation becomes costly.

The present study introduces a virtual-wall model that can significantly reduce the computational time when dealing with the fluid-wall interactions while achieving the same results as the atomic wall model. The remainder of this paper is organized as follows. Section 2 introduces the virtual-wall model. Section 3 and Section 4 describe the application of the model to the stationary wall gravitation-driven Poiseuille flow and to the moving wall shear-driven Couette flow, respectively. Section 5 presents the model analysis. Finally, Section 6 elaborates on the conclusions of the study.

## 2. Virtual-Wall Model

A crystal structure is a unique arrangement of atoms or molecules in a crystalline solid [28]. It describes a highly ordered structure as a result of the intrinsic nature of molecules to form symmetric patterns. A crystal structure can be regarded as an infinitely repeating array of 3D “boxes” known as unit cells. Most metals and many other solids have unit cell structures described as body-centered cubic (BCC), face-centered cubic (FCC), or hexagonal close packed. For example, the unit cell structure of platinum is FCC.

**Figure 1:**
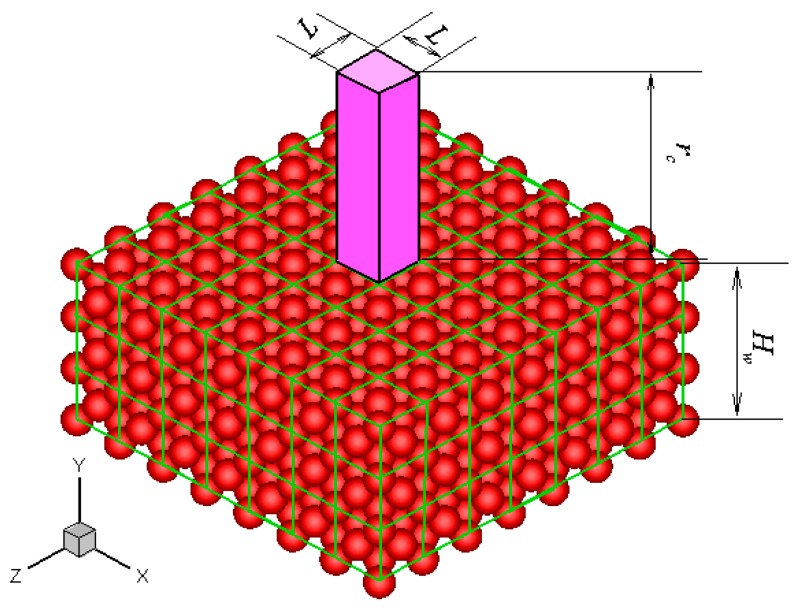
Schematic of the FCC atomic wall and the unit cuboid domain above the wall surface.

Without losing generality, a platinum wall is considered, and the FCC structure is used. The infinite wall is along the *xz* plane, as illustrated in Figure 1. The thickness of the wall, *H_w_*, is set to be larger than the fluid-wall interactional cut-off radius, *r_c_*. The interactions between fluid-fluid and fluid-wall molecules are described by truncated and shifted Lennard-Jones potential, which is given as follows:

(1) $$V\left(r_{ij}\right)=\left\{ \begin{array}{ll} 4\varepsilon \left[{\left(\frac{\sigma }{r_{ij}}\right)}^{12}-{\left(\frac{\sigma }{r_{ij}}\right)}^{6}-{\left(\frac{\sigma }{r_{c}}\right)}^{12}+{\left(\frac{\sigma }{r_{c}}\right)}^{6}\right] & r_{ij}\leq r_{c}\\ 0 & r_{ij}>r_{c} \end{array}\right.$$

where *r_ij_* is the intermolecular distance between molecules *i* and *j*, *ε* is the depth of the potential well, *σ* is the molecular diameter, and *r_c_* is the cut-off radius. Argon was employed as the fluid molecule in this study. The diameter, *σ*_Ar_, and potential depth *ε*_Ar_ of argon molecule are 0.3405 nm and 119.8 k_B_, respectively. For the argon-platinum molecular interaction, *σ*_Ar-Pt_ is 0.3085 nm and *ε*_Ar-Pt_ is 64.8 k_B_ [29]. *r_c_* was chosen to be 0.851 nm, which is approximately equal to 2.5*σ*_Ar_.

**Figure 2:**
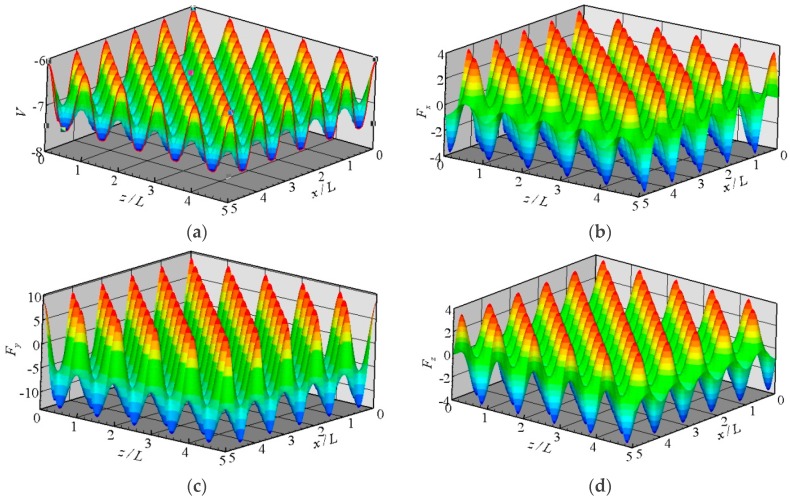
Distributions of the potential and forces at the plane *y* = 0.3405 nm above the wall surface, (**a**) potential; (**b**) *F_x_*; (**c**) *F_y_*; and (**d**) *F_z_*.

The potential between a fluid molecule and the fixed FCC wall molecules depends on the position of the fluid molecule. The distribution of the potential at a plane that is 0.3405 nm above the wall surface is shown in Figure 2a. The location of the wall surface is defined at the center of the first row of the wall molecules facing the fluid. The potential is periodic in the *x* and *z* directions as a result of the repeating array of unit cells, as illustrated in Figure 1.

The force that acts on a fluid molecule from the wall molecules can be obtained as,

(2) $$F=\sum_{i}^{N}\left(-\nabla V_{i}\right),$$

where *N* is the number of wall molecules that interact with the fluid molecule. The distributions of three force components at the plane *y* = 0.3405 nm are also shown in Figure 2. These forces are all periodic in the *x* and *z* directions. The potential and forces of a fluid molecule located at (*x*, *y*, *z*) are exactly the same as the potential and forces of the same molecule located at (*x* + *iL*, *y*, *z* + *kL*), where *i* and *k* are integers, and *L* is the lattice constant. If the force distributions in the unit cuboid domain (*L* × *r_c_* × *L*) in Figure 1 are given, then the force anywhere else can be determined. This feature is the core concept of the virtual-wall model.

The unit cuboid domain is divided into *N_x_* × *N_y_* × *N_z_* bins. The forces on a fluid molecule located at each bin center from the wall molecules are calculated and stored in an *N_x_* × *N_y_* × *N_z_* array in the memory. If the distance of a fluid molecule to the wall surface is larger than *r_c_*, then no fluid-wall interaction is calculated and the fluid molecule only interacts with other fluid molecules. Otherwise, the fluid molecule interacts with the wall molecules, and other fluid molecules. The intermolecular force between this fluid molecule and the wall molecules can be determined by mapping the location of the fluid molecule to the unit cuboid domain and searching for the bin to which this location belongs. Linear interpolation is used in the simulation to obtain the accurate intermolecular force at the fluid molecular location. This virtual-wall model avoids calculating excessive fluid-wall interactions. Although an additional force array is used in the virtual-wall model, the computational time and memory can be significantly saved.

In studying the stress tensor of fluids in MD simulation, the virial components [30] between the interactional fluid-wall molecules must also be calculated and stored. The reason for this is that the virial term dominates the stress tensor in the near-wall region [15].

## 3. Stationary Wall Poiseuille Flow

The open source MD code called Large-scale Atomic/Molecular Massively Parallel Simulator (LAMMPS) [31], which was developed by Sandia National Laboratories, was used in this study. The virtual-wall model is embedded into the LAMMPS code. The virtual wall MD simulation is initially applied to the stationary wall gravitation-driven Poiseuille flow. The results are validated using the corresponding conventional atomic wall MD simulation.

In the atomic wall simulation, the channel is 40.0 nm × 15.33 nm × 40.0 nm in *x*, *y*, and *z* directions. The channel is confined between two platinum walls. The wall molecules are frozen. The thickness of the wall is 0.98 nm, which is larger than *r_c_*. The lattice constant of the FCC platinum lattice is 0.392 nm. The simulation box is periodic in the *x* and *z* directions. The initial fluid density is 8.4 kg/m^3^ and the fluid temperature is 300 K. The mean free path of argon is approximately 15.33 nm. The Knudsen number, *Kn*, according to the channel height is 1.0. Thus, the gas flow is in the transition regime. In our simulations, 3087 gas molecules and 105,063 wall molecules were employed. A force of 0.008*ε*_Ar_*/σ*_Ar_ acted on each fluid molecule to drive the gas flow [32]. Bins of 40 × 100 × 40 were used. In the virtual wall simulation, all parameters were set to be the same as those in the atomic wall simulation, except that no wall molecules were employed.

The neighbor-list method was used to calculate the force between atoms and the velocity-Verlet algorithm was used to integrate the equations of motion in micro canonical ensemble [31]. The time step in the simulation was 10.8 fs. Macro quantities, such as density, velocity, temperature and pressure were sampled from 8 million time steps in 250 bins in the *y* direction, after 2 million steps for equilibrium. Only thermal velocities were used to compute the temperature and pressure. The direct rescaling thermostat method [33] was employed to maintain the fluid at the desired temperature.

**Figure 3:**
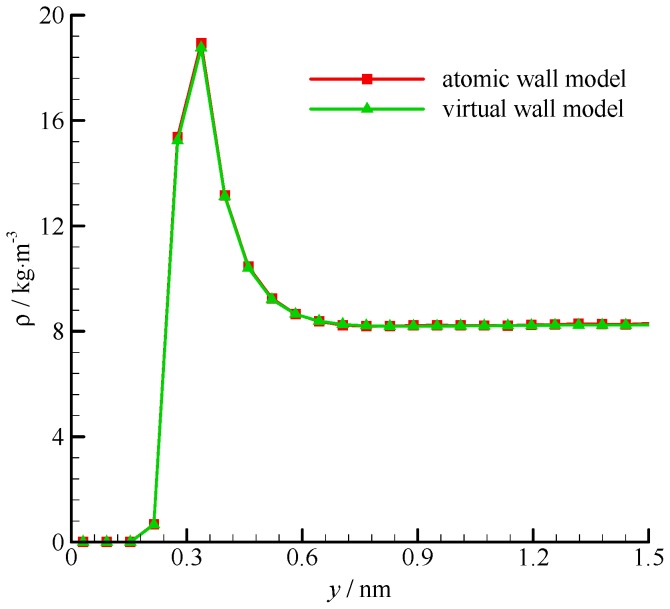
Fluid density profiles of the atomic and virtual wall MD simulations near the wall in Poiseuille flow, using the dimensional wall distance.

The computational time of the atomic wall simulation on one processor of an Intel i5-2500 CPU was 97.3 h. In the virtual wall simulation, the computational time was 1.14 h. The fluid density profiles of the atomic and virtual wall MD simulations within 1.5 nm from the wall surface are shown in Figure 3. Fluid densities are constant in the channel, except near the wall, where a single density buildup was observed [34]. The residence time of fluid molecules at this position was longer than those of any other positions. This phenomenon was due to the potential minimum of the wall molecules. The fluid density within 0.5*σ*_Ar_ (0.17 nm) from the wall was zero because the wall molecules are positioned at *y* = 0 and the repulsive force is too large for the fluid molecules to penetrate to this region. The atomic and virtual wall MD simulations present exactly the same density profile.

**Figure 4:**
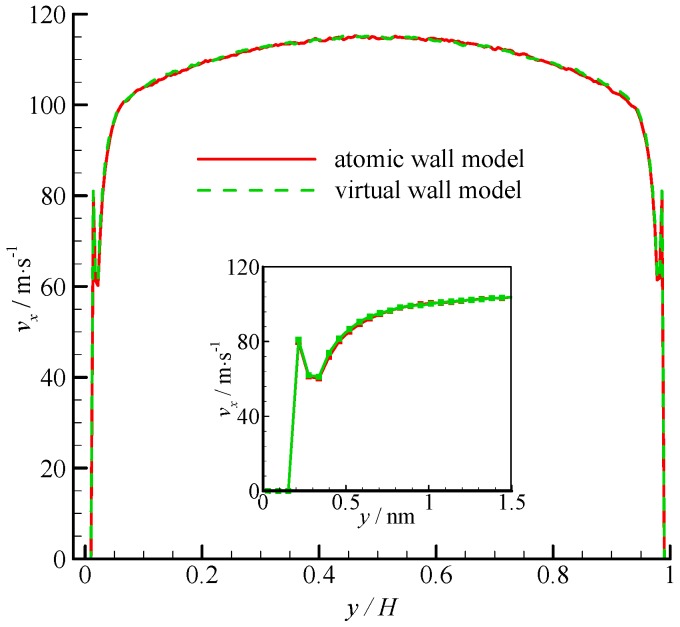
Velocity profiles of the atomic and virtual wall MD simulations across the channel in Poiseuille flow. The non-dimensional wall distance (non-dimensionalized by the channel height *H*) is used in large figure, and the dimensional wall distance is used in the zoomed-in figure.

The velocity profiles of the two simulations are compared in Figure 4. Parabolic velocity profiles can be found in the bulk of the channel in both simulations. The velocity decreases rapidly when approaching the wall. The zoomed in velocity profile within 1.5 nm from the wall surface is also shown in Figure 4. Significant velocity slips on the wall can be observed. Given that the fluid molecules cannot penetrate the bins adjacent to the wall, the fluid velocities are zero. A velocity peak occurs at the near wall region. The velocity peak always exists for different wall unit cell structures, gas densities, and integration timesteps. This phenomenon may be due to the small density in this bin. As shown in Figure 4, the velocity of the virtual-wall model agrees well with that of the atomic wall model.

## 4. Moving Wall Couette Flow

The virtual-wall model was subsequently applied to Couette flow. All the geometries and parameters were chosen to be the same as those of Poiseuille flow in Section 3, except that no additional forces acted on each fluid molecule, but the upper wall moved at a constant velocity. The virtual-wall model was developed to account for the moving wall effect.

**Figure 5:**
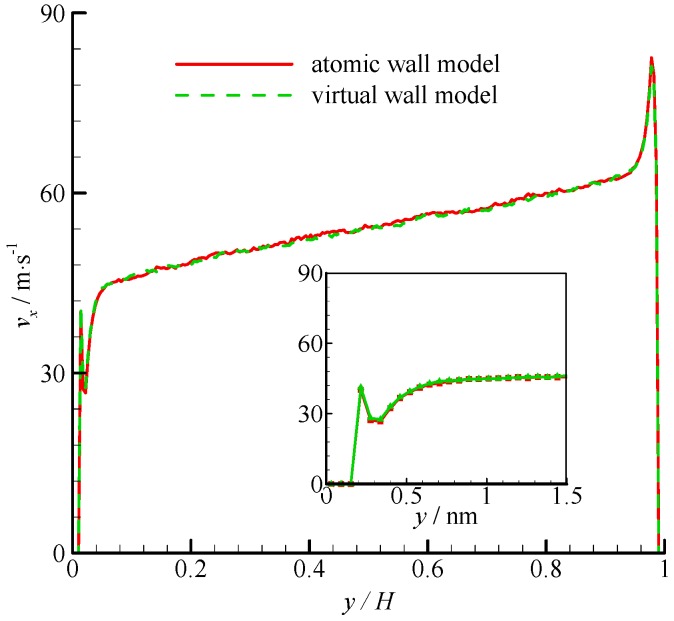
Velocity profiles of the atomic and virtual wall MD simulations across the channel in Couette flow. The non-dimensional wall distance (non-dimensionalized by the channel height *H*) is used in large figure, and the dimensional wall distance is used in the zoomed-in figure.

The density profiles of Couette flow obtained from the atomic and virtual wall MD simulations were the same as those of Poiseuille flow. They are not presented in this paper for brevity. The velocity profiles of these two simulations are compared in Figure 5. The velocity profile is linear in the bulk region of the channel. In the near-wall region, significant velocity slip can be found at the wall. The zoomed in velocity profiles within 1.5 nm from the wall are also shown in Figure 5. The two simulations agree well each other.

**Figure 6:**
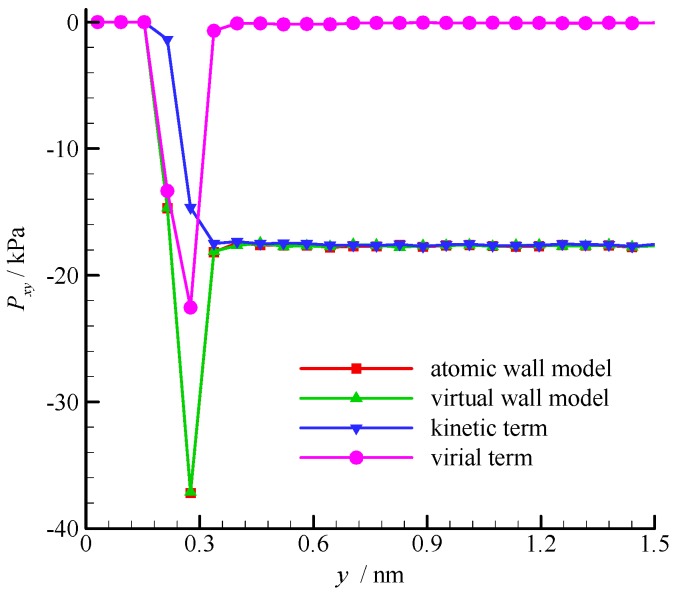
Shear stress profiles of the atomic and virtual wall MD simulations near the wall in Couette flow, using the dimensional wall distance.

The fluid shear stress profiles near the wall surface are compared in Figure 6. The shear stress is constant in the bulk region of the channel, but large fluctuation exists near the wall. According to the pressure tensor decomposition proposed by Irving and Kirkwood [30], the shear stress is composed of kinetic and virial terms. The kinetic term depends on the molecular square velocity, whereas the virial term relies on the pairwise interactions between molecules. These two terms of the virtual wall molecular dynamics calculation are also shown in Figure 6. The virial term vanishes in the bulk region of the channel because the average intermolecular distance (*r_ave_* = 2.0 nm when ρ = 8.4 kg/m^3^) is larger than *r_c_*, and the interaction between fluid molecules is small. Hence, the kinetic term dominates the shear stress in the bulk region. In the near-wall region, the fluid-wall interaction leads to a large virial term. Therefore, the virial term dominates the shear stress. The results of the atomic and virtual wall MD simulations present excellent agreement.

## 5. Model Analysis

### 5.1. Computational Time Reduction Effect

The proposed virtual-wall model reduces the computational time when dealing with the wall in the MD simulation. For example, we showed the computational time reduction for Poiseuille flow at different fluid densities in a 40 × 15.33 × 40 nm channel confined between two 0.98 nm FCC platinum walls. In all simulations, *r_c_* equal to 2.5*σ*_Ar_, and 250 bins were used. To compare the computational time, each simulation ran 10,000 timesteps on one processor of an Intel i5-2500 CPU. The computation parameters and results are shown in Table 1.

The numbers of wall molecules are the same in the nine cases. By contrast, the number of fluid molecules varies with the fluid density, but the channel geometry is fixed. When the fluid density is small, e.g., ρ = 1.68 kg/m^3^ in case 1, the number of fluid molecules is less than that of the wall molecules. In the atomic wall model, most computation is consumed by the interactions between the wall-fluid molecules. However, these interactions between the wall-fluid molecules are replaced by pretabulated mappings. As a result, the computational time of the atomic wall simulation is 278.77 s, whereas that of the virtual wall simulation is only 1.03 s. The increase in fluid density increases the number of fluid molecules and reduces the time reduction ratio. However, although ρ = 840.3 kg/m^3^ in case 9, the computational time of the virtual-wall model is still 1.47 times less than that of the atomic wall model.

**Figure 7:**
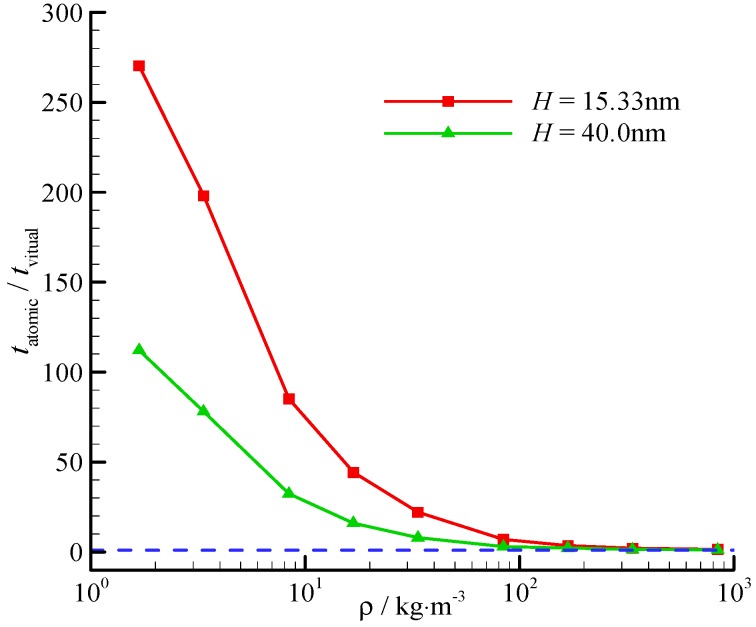
Computational time reduction ratio of the virtual-wall model. The blue dashed line represents for the time reduction ratio is 1.

The computational time reduction ratio also varies with the channel height. The time reduction ratios at two different channel heights (*H* = 15.33, 40.0 nm) are shown in Figure 7. The time reduces significantly at a small channel height, and the fluid-surface interaction is significant.

### 5.2. Determination of Bin Size

The size of unit cuboid domain (*L* × *r_c_* × *L*), as illustrated in Figure 1, is determined by the wall unit cell structure, *L*, and the fluid-wall interactional cut-off radius, *r_c_*. The unit cuboid domain is divided into *N_x_* × *N_y_* × *N_z_* bins. In the virtual-wall model, all fluid molecules located in the same bin (with bin size $dx=L/N_{x}$, $dy=r_{c}/N_{y}$, and $dz=L/N_{z}$) share the same force value (*F_xi_*, *F_yj_*, *F_zk_*). The deviation of the bin value (*F_xi_*, *F_yj_*, *F_zk_*) from the molecular actual force (*F_x_*, *F_y_*, *F_z_*) increases with the increase in bin size. When the bin is large, the results of the virtual-wall model may deviate from those of the atomic wall model. The number of bins is large in the case of a small bin size, thereby requiring large computational memory and time. An appropriate bin size must be determined in the virtual-wall model. For convenience, a simple cubic lattice is used. The lattice constant is 1*σ*_Ar_ and the fluid-wall interactional cut-off radius is 2.5*σ*_Ar_. Four bin sizes (*dx* = *dy* = *dz* = 0.1, 0.05, 0.025, 0.0125*σ*_Ar_) are investigated.

**Figure 8:**
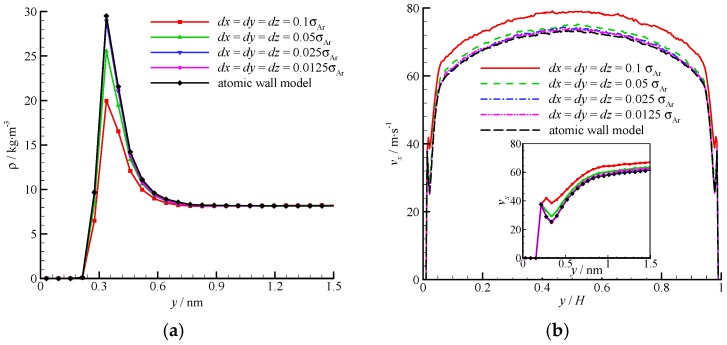
Comparison of the density and velocity profiles at different bin sizes, (**a**) density profile and (**b**) velocity profile. The non-dimensional wall distance (non-dimensionalized by the channel height *H*) is used in large figure, and the dimensional wall distance is used in the zoomed-in figure.

The density and velocity profiles of the virtual wall MD simulations at the four bin sizes are compared in Figure 8. With the decrease in bin size, the density and velocity show steady profiles. The results of the virtual wall simulation with the bin size of 0.025*σ*_Ar_ agree well with those of 0.0125*σ*_Ar_. Therefore, *dx* = *dy* = *dz* = 0.025*σ*_Ar_ is adequate in the virtual-wall model.

### 5.3. Effect of the Fluid-Wall Interactional Cut-Off Radius

The interactional cut-off radius is used in MD simulations to reduce the computational time. When the distance between two molecules is larger than the cut-off radius, the potential between the two molecules is small and neglected. In equilibrium fluid flows, the fluid is isotropic and the fluid molecules surrounding the sphere domain with radius *r_c_* are symmetrical. A small *r_c_* may thus be considered acceptable. However, when a fluid molecule is located near the wall, the molecules outside the sphere domain with radius *r_c_* are not symmetrical. Considerable attraction from the wall molecules will act on the fluid molecule because the attraction from the gas side is negligible. Different cut-off radii may lead to different fluid flow characteristics in nano-scale channels. More wall molecules must be used in the simulation, to guarantee that *H_w_* is larger than *r_c_*. *r_c_* = 2.5*σ*_Ar_ was used in previous sections because the computational time increases rapidly with *r_c_*, e.g., in case 3 in Section 5.1. When *r_c_* = 5*σ*_Ar_, 210,125 wall molecules must be used in the atomic wall simulation and the computational time for 10,000 timesteps is 3006.04 s. This result is 8.6 times larger than that when *r_c_* = 2.5*σ*_Ar_. In the virtual wall simulation, no wall atoms are used. Only the array size in the *y* direction, *N_y_*, needs to be enlarged to account for the large cut-off radius. The computational time does not change with the fluid-wall interactional cut-off radius.

**Figure 9:**
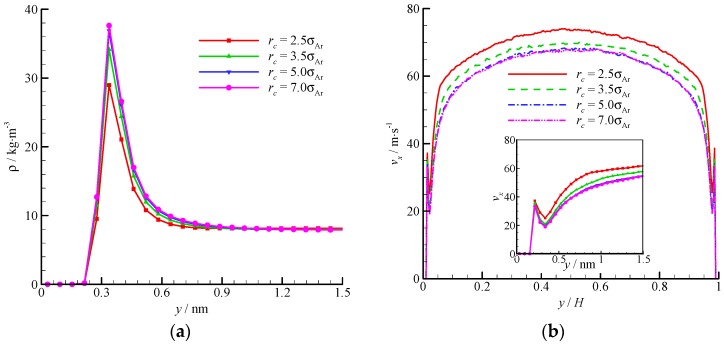
Comparison of density and velocity profiles at different interactional cut-off radii, (**a**) density profile and (**b**) velocity profile. The non-dimensional wall distance (non-dimensionalized by the channel height *H*) is used in large figure, and the dimensional wall distance is used in the zoomed-in figure.

The density and velocity profiles across the channel at different interactional cut-off radii are shown in Figure 9. Four cut-off radii (*r_c_* = 2.5, 3.5, 5, and 7*σ*_Ar_) were studied. A large cut-off radius leads to a strong attraction. Thus, considerable fluid molecules are trapped by the wall and accumulate near the wall. The density-buildup phenomenon near the wall is significant, and the streamwise velocity is small at a large cut-off radius. A *r_c_* = 2.5*σ*_Ar_ is inadequate when dealing with the fluid-wall interaction, and a larger cut-off radius (e.g., *r_c_* = 5*σ*_Ar_) must be adopted. The virtual-wall model shows its superiority in the MD simulation with a large cut-off radius.

### 5.4. Other Applications

**Figure 10:**
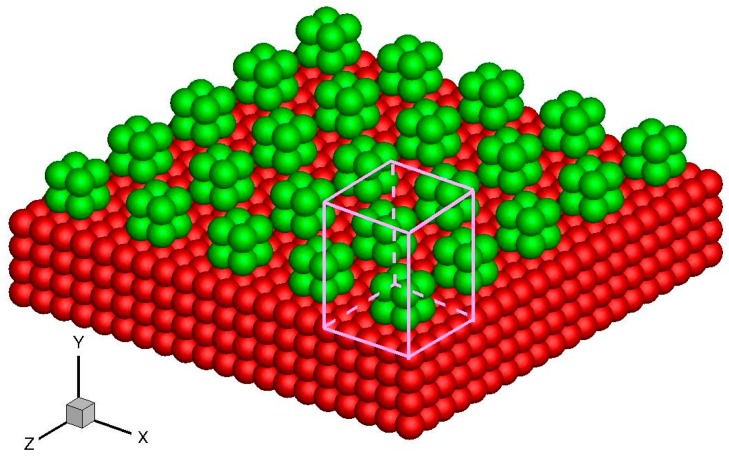
Schematics of the rough wall and unit cuboid domain above the wall surface. The red atoms are smooth wall atoms and the green atoms are roughness elements.

Apart from the above-mentioned smooth stationary and moving walls, periodically structured walls, such as a smooth wall with periodic roughness elements and single-walled or multi-walled carbon nanotubes, can also be modeled by the virtual-wall model. These walls can be considered an infinitely repeating array of unit structures. For example, in the rough wall simulation in Figure 10, the roughness element is 2*σ*_Ar_ × 2*σ*_Ar_ × 2*σ*_Ar_ and the distance between two elements is 4*σ*_Ar_. Hence, the rough wall can be an infinitely repeating unit structure with dimensions 4*σ*_Ar_ × 4*σ*_Ar_ in the *x* and *z* directions. The force on a fluid molecule from the wall molecules is periodic in the *x* and *z* directions. A unit cuboid domain, also illustrated in Figure 10, can therefore be introduced. If the interactional cut-off radius is 2.5*σ*_Ar_, then the size of the unit cuboid domain is 4*σ*_Ar_ × 4.5*σ*_Ar_ × 4*σ*_Ar_. The unit cuboid domain is divided into bins of 125 × 150 × 125, and the forces in each bin are calculated and stored in the memory. This force array is later invoked in the MD simulation.

The Poiseuille flow between two rough walls was also studied. The gas density was 8.4 kg/m^3^ and the channel height was 15.33 nm. A simple cubic lattice was used. The computational time of the virtual-wall model for 10,000 timesteps was 4.56 s, which is 60.4 times less than that of the atomic wall model, i.e., 275.29 s.

**Figure 11:**
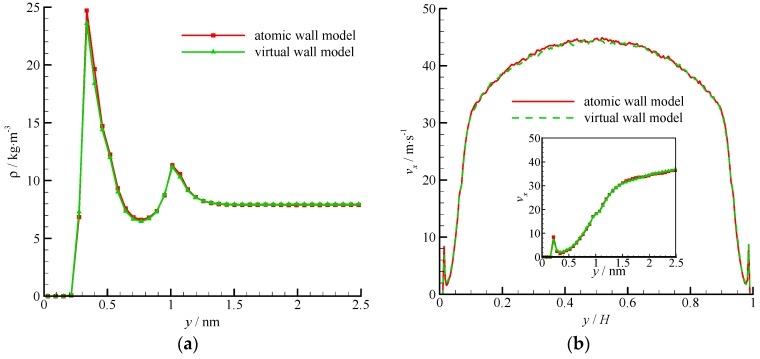
Density and velocity profiles of the atomic and virtual wall MD simulations in Poiseuille flow with rough wall, (**a**) density profile and (**b**) velocity profile. The non-dimensional wall distance (non-dimensionalized by the channel height *H*) is used in large figure, and the dimensional wall distance is used in the zoomed-in figure.

The density and velocity profiles across the channel of the virtual wall simulation in comparison with the corresponding results of the atomic wall simulation are shown in Figure 11. The results of the virtual-wall model agree well with those of the atomic wall model. Two density peaks near the roughness wall can be found in the density profile. The distance between the two density peaks is approximately 2*σ*_Ar_, which is the height of the roughness element. The wall force penetration depth is larger and the average velocity is smaller than those of the smooth wall flow.

## 6. Conclusions

A large number of molecules are usually required in a MD simulation to model the atomic wall in nano-scale confined fluid flows. This drawback is especially fatal in gas flows, where the number of wall molecules can be several hundred times larger than that of the gas molecules. The virtual-wall model proposed in this study reduces the computational time when dealing with fluid-wall molecular interactions in the MD simulation.

The virtual-wall model was applied to the smooth stationary wall Poiseuille flow and moving wall Couette flow. The flow properties were compared with those of traditional atomic wall simulations. The two results agree well with each other, thereby indicating that the virtual-wall model works effectively in the MD simulation. The virtual-wall model drastically reduced the computational time. The time reduction effect is significant at small fluid density and channel height. The bin size and interactional cut-off radius in the virtual-wall model were also discussed. A bin size of 0.025*σ*_Ar_ is considered to be adequate, and *r_c_* = 5.0*σ*_Ar_ is suggested in nano-scale confined flows.

The virtual-wall model could also be used to model periodically structured walls, such as a rough wall and single-walled or multi-walled carbon nanotubes, apart from smooth stationary and moving walls. However, given that the wall molecules are frozen at their lattice, the virtual-wall model cannot be used to study heat transfer characteristics. Nevertheless, this model can be developed in future work.

## Figures and Tables

**Table 1:** Comparison of computational times between the atomic and virtual-wall models.

Case	Fluid Density (kg/m^3^)	Mean Free Path (nm)	*Kn*	Fluid Molecules	Wall Molecules	Computation Time (Atomic Wall Model) (s)	Computation Time (Virtual-Wall Model) (s)	Time Reduction Ratio
1	1.68	76.64	5	576	105,063	278.77	1.03	270.27
2	3.36	38.32	2.5	1125	105,063	297.68	1.50	198.12
3	8.4	15.33	1	3087	105,063	350.35	4.12	85.08
4	16.8	7.66	0.5	6084	105,063	391.58	8.86	44.22
5	33.6	3.83	0.25	11,264	105,063	423.22	19.17	22.08
6	84.0	1.53	0.1	30,976	105,063	575.98	81.94	7.03
7	168.1	0.77	0.05	60,500	105,063	838.66	235.34	3.56
8	336.1	0.38	0.025	119,025	105,063	1576.99	764.97	2.06
9	840.3	0.15	0.01	294,066	105,063	5195.41	3531.47	1.47
